# Identification of nonsense-mediated mRNA decay pathway as a critical regulator of p53 isoform β

**DOI:** 10.1038/s41598-017-17283-4

**Published:** 2017-12-13

**Authors:** Lauren E. Cowen, Yi Tang

**Affiliations:** Department of Regenerative and Cancer Cell Biology, Albany Medical College 47 New Scotland Ave., Albany, NY 12208 USA

## Abstract

Human *TP53* gene encodes the tumor suppressor p53 and, via alternative splicing, the p53β and γ isoforms. Numerous studies have shown that p53β/γ can modulate p53 functions and are critically involved in regulation of cellular response to stress conditions. However, it is not fully understood how the β and γ isoforms are regulated following splicing. Using gene targeting and RNAi, we showed that depletion of the nonsense-mediated mRNA decay (NMD) factor SMG7 or UPF1 significantly induced p53β but had minimal effect on p53γ. Sequence analysis reveals the presence of unique features – key hallmarks of NMD targets in the p53β transcript, which was further confirmed in NMD reporter gene assays. By manipulating splicing components, we found that NMD activities are crucial to control p53β levels under conditions that favor its splicing. Our data demonstrate that the NMD and alternative splicing pathways regulate p53β in a synergistic manner, and NMD plays a critical role in the determination of the p53β following its splicing. As aberrant p53β expression and dysfunctional NMD are both implicated in cancers, our studies may provide a novel insight into the regulation of p53β in tumorigenic settings.

## Introduction

Transcription factor p53 is known for its role in regulating a complex gene network involving the induction of tumor suppressive pathways including senescence, cell cycle arrest and apoptosis^1,2^, which can be modulated by its isoforms^3–5^. The *TP53* gene encodes several distinct isoforms which can function both redundantly to p53 (also known as p53α) and in a dominant-negative manner^3–5^. The p53 isoforms β and γ which form as a result of alternative splicing, are expressed at relatively low levels and reside predominantly in the nucleus, allowing them to bind differentially to p53 target promoters as potent activators of p53α activity^3–7^. Both isoforms have been found to be dysregulated in cancers^8–14^, and implicated in contributing to the p53α tumor suppressive function^11,15^. Typically, the loss of p53 isoforms β/γ expression correlates with cancer progression, whereas maintained expression correlates with an improved clinical outcome (i.e. increased survival and chemo-sensitivity, decreased tumor size and recurrence etc.)^9,11,16^. Moreover, elevated expression of the p53 β/γ isoforms has the potential to counteract the negative clinical implications of some *TP53* mutations suggesting a compensatory mechanism or redundant function to p53α^9^. The discovery of dysregulation of p53 isoforms in cancers may also shed light on the challenges behind linking *TP53* status to clinical outcome which previously, had been difficult to correlate^5^. In summary, investigating p53β/γ isoform expression and their regulation may provide critical insights into predicting clinical outcomes and improving therapeutic approaches.

In light of these clinical observations, extensive efforts have recently been made to understand how p53 isoforms including p53β and p53γ are regulated in human cancer^5^. A critical mechanism for regulation of the β and γ isoforms is alternative splicing, which allows for the initial generation of the p53β/γ transcripts, and has been proposed as a novel therapeutic target. Alternative splicing is known to be regulated at the level of serine/arginine-rich splicing factors (SRSFs), which can be preferentially recruited to facilitate differential splicing of various isoforms^17^. For example, it was observed that a loss of splicing factor SRSF3 resulted in an upregulation of p53β expression^17^. Furthermore, suppression of SRSF1, which  favors splicing p53α, promotes p53β/γ splicing^18^. Additionally, SRSF7 can be regulated following gamma irradiation, ultimately resulting in the induction of alternative splicing of p53β in response to cellular stressors like DNA damage^19^. Taken together, this demonstrates a critical role for various SRSFs in regulating p53 isoform alternative splicing however, it remains unknown how the p53β/γ mRNAs are further post transcriptionally processed following their splicing.

The nonsense-mediated mRNA decay (NMD) pathway, initially thought as a quality control mechanism for elimination of aberrant mRNAs, is now appreciated as a diverse regulatory network for mRNA cellular fate throughout development and in response to cellular stressors, and is critical for maintaining mRNA homeostasis^20,21^. Disruption of the NMD pathway has been linked with numerous genetic diseases^20^, as well as cancer, likely as a result of dysregulation of tumor suppressors/oncogenes^12,21–24^. NMD is carried out by a complex of RNA binding proteins, mainly the UPF1 (regulator of nonsense transcripts 1 (RENT1)^25–29^) protein which is recruited to mRNA during translation if a premature termination codon (PTC)^20,30,31^ is found and initiates recruitment of SMG (suppressor for morphological effect on genetalia) family proteins (SMG5, SMG6, and SMG7) and other cofactors to the mRNA for decapping, deadenyation, and ultimately mRNA decay^20^. SMG7, a critical NMD factor^32,33^, was shown by our group to be a novel regulator of p53α protein stability under DNA damage conditions^34^. Here, we sought to investigate whether SMG7 can regulate p53 via its NMD activity. Despite p53β having key hallmarks of a NMD target, its regulation via the NMD pathway has been largely unexplored. Our studies reveal an additional mechanism by which SMG7 can regulate p53 as well as demonstrate a novel function of the NMD pathway in regulation of p53β cellular fate.

## Results

### Loss of *SMG7* results in NMD deficiency and upregulation of p53β

**Figure 1:**
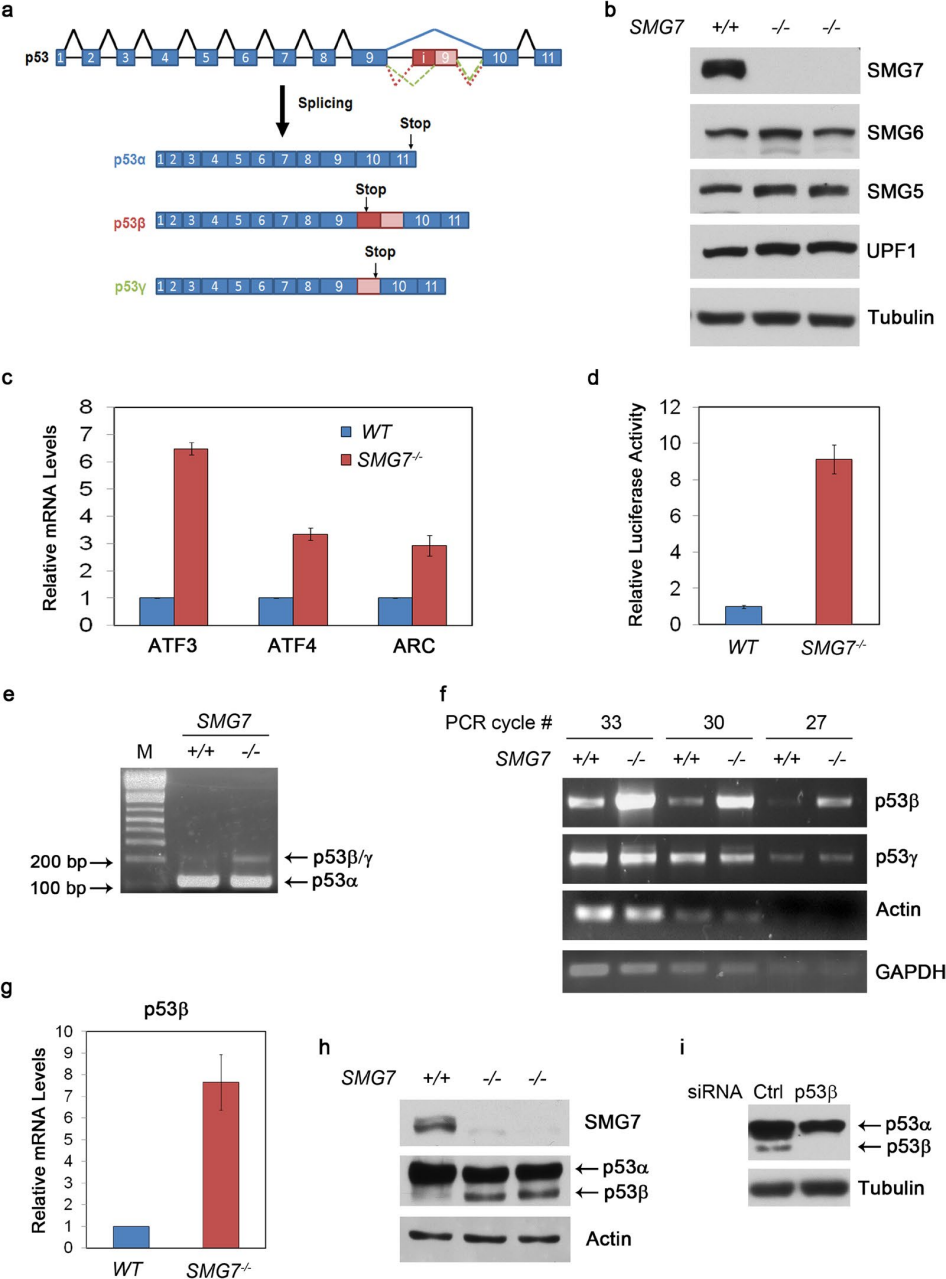
Loss of *SMG7* results in NMD deficiency and upregulation of p53β. **(a)** Scheme depicting the splicing of the p53 transcript into p53 α, β, and γ isoforms. **(b)** Characterization of HCT116 wild-type and *SMG7* ^−/−^ cells (Clone #1 & #2^34^) via western blot analysis specifically NMD core factor expression. **(c)** qPCR analysis of known NMD targets (ATF3, ATF4, & ARC, Supplemental Table 2) in HCT116 WT and *SMG7* ^−/−^ cells, (n = 3). **(d)** Assessment of NMD function via NMD luciferase assay as previously described (pCL-Neo β-globin WT Renilla, pCL- Neo β-globin NT39 Mutant Renilla, and pCL- Neo Firefly)^35^. Persistence of luciferase indicates the absence of NMD decay of the transcript in HCT116 WT and *SMG7* ^−/−^ cells. *Renilla* Luciferase signals were normalized to *Firefly* Luciferase controls, (n = 6). **(e)** RT-PCR for amplification of p53 isoform mRNA using primers spanning exon 9 and exon 10 (Supplemental Table 2). Inclusion of intron i9 (β or γ) would generate PCR fragments of larger sizes. **(f)** Nested semi-quantitative PCR (Supplemental Table 2) of p53 isoforms as previously described^36^ in HCT116 WT and *SMG7* ^−/−^ cells. Samples were run at various cycle numbers (33, 30, 27) to ensure linear amplification. **(g)** qPCR analysis of p53β levels in HCT116 WT and *SMG7* ^−/−^ cells (Supplementary Table 2), (n = 8). **(h)** Western blot analysis of HCT116 WT and *SMG7* ^−/−^ cells for p53 isoform expression, with p53 antibody (α, β, or γ) β-actin. **(i)** Identification of the lower p53 protein band as p53β. *SMG7* ^−/−^ cells were transfected with p53β specific siRNA (Supplementary Table 1,^18^). Western blot analysis of p53 was carried out as in **(h)**.

In our recent study, we show that the NMD factor SMG7 plays a critical role in the control of p53 protein stability following DNA damage^34^. Here, we investigate whether SMG7 can regulate p53 through the nonsense-mediated mRNA decay (NMD) pathway, as p53 isoforms β and γ are derived from alternative splicing of intron 9 and each contain a premature stop codon (PTC) before the last intron^3^, a common feature of NMD targets (Fig. 1a). To this end, we utilized the human colorectal cancer HCT116 cells and the isogenic *SMG7-*null cells generated in our previous study^34^. As shown in two independent clones (Fig. 1b), deletion of both *SMG7* alleles resulted in complete loss of SMG7 proteins and had minimal effect on the expression of other key NMD factors including SMG5, SMG6 and UPF1. As expected, the *SMG7-*null cells exhibited deficient NMD activities as indicated by the induction of several known NMD targets (ATF3, ATF4 and ARC^20^; Fig. 1c) and the enhanced luciferase activities in the NMD reporter assays^35^ (Fig. 1d and Supplementary Fig. S1).

To examine p53β/γ, we performed PCR using primers proximally flanking intron 9, which allows the detection of all C-terminally spliced isoforms. As shown in Fig. 1e, besides p53α (the lower band, 115 bp) a DNA fragment of more than 200 bp was strongly amplified only from the *SMG7-*null cells, suggesting the loss of SMG7 may induce p53β/γ expression. To corroborate this, we examined the p53β/γ full-length transcripts using the nested semi-quantitative PCR approach^36^. Interestingly, we found that loss of SMG7 had minimal effect on p53γ but strongly induced p53β (Fig. 1f), which was also demonstrated using qPCR (Fig. 1g and Supplementary Fig. S1). By western blot analysis of two independent HCT116 *SMG7-*null clones, we observed a robust induction of p53 protein bands at a lower molecular weight (Fig. 1h). Using a p53β specific siRNA (Supplementary Table 1;^18^), we were able to identify these bands as p53β (Fig. 1i). Furthermore, as p53 can form several N-terminal truncated isoforms which also include β forms, we also assessed Δ133 p53 expression via nested PCR^3^. Our results indicate that although the overall expression of the full-length Δ133 p53 transcript is not increased, the expression of the Δ133 p53β transcript is increased in the *SMG7* ^−/−^ cells (Supplementary Fig. S1) suggesting NMD can regulate other N-terminal truncated p53 isoforms which contain the i9β region. Taken together, these results show a critical role of SMG7-mediated NMD in the suppression of p53β at the mRNA level as well as the protein level.

### SMG7 differentially regulates p53β and p53γ

**Figure 2:**
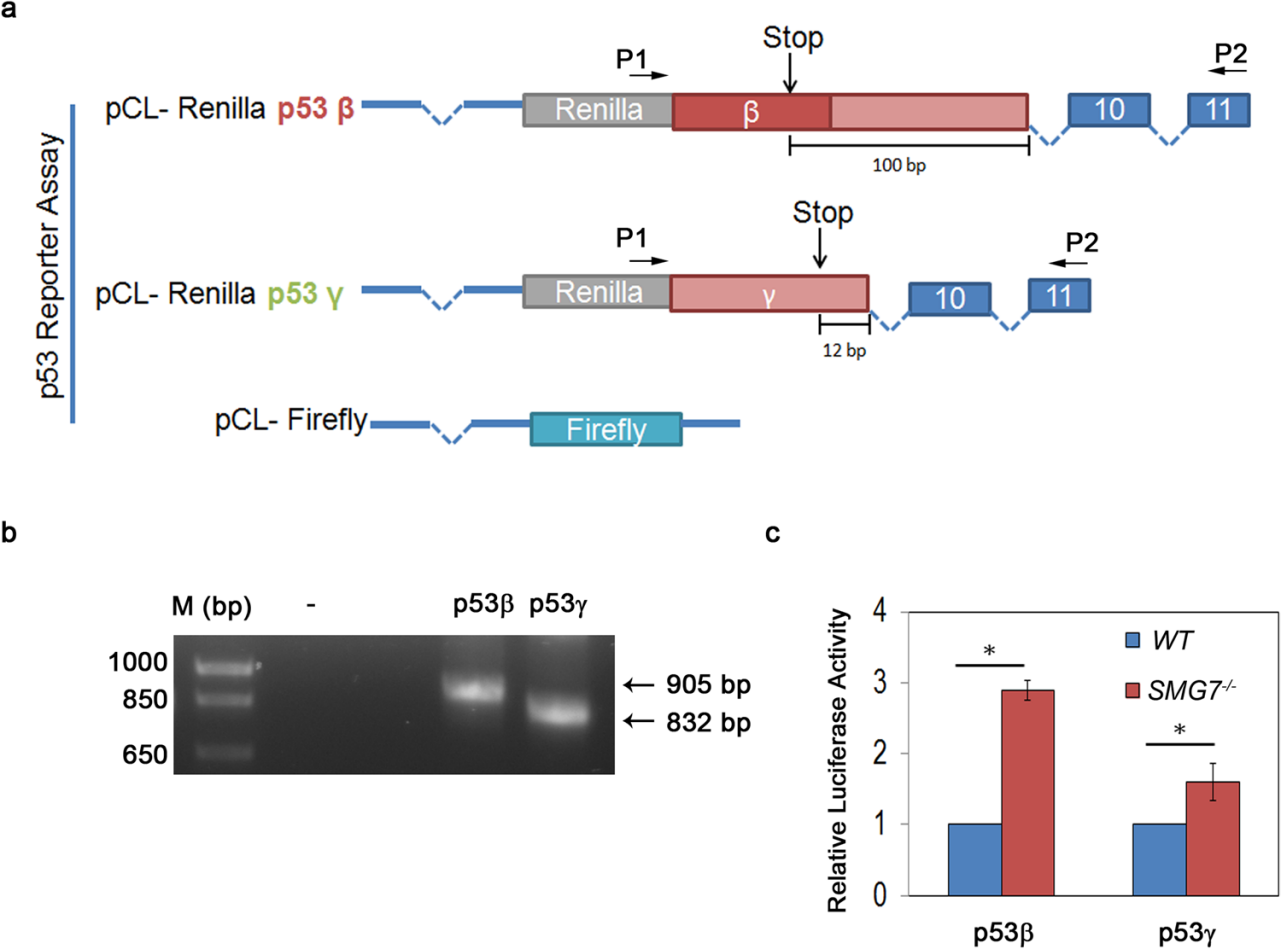
SMG7 differentially regulates p53β and p53γ. **(a)** Scheme depicting the NMD reporter constructs for the p53 isoforms β and γ. The p53 genomic DNA region spanning from the intron-9-derived exon to the last exon 11 was cloned into the pCL-Renilla backbone via multiple cloning sites (Supplemental Table 2). We confirmed in-frame insertion via diagnostic restriction enzyme digestion and sequencing. Further cloning details are available upon request. **(b)** RT-PCR confirmation of appropriate splicing of p53 reporter constructs at established canonical sites. Arrows indicate bands of expected size for complete and appropriate splicing (Supplemental Table 2). **(c)** Analysis of p53 isoform expression via the luciferase reporter assays. Persistence of luciferase indicates the absence of NMD decay of the transcripts, p = 0.003 (p53β), p = 0.048 (p53γ). Statistical analysis was carried out using students two tailed, unpaired, *t*-test; p < 0.05, SEM, n = 6. Error bars are utilized to represent SEM.

Loss of *SMG7* induces p53β but has minimal effect on p53γ expression, suggesting that p53γ is not targeted by NMD. This is indeed intriguing given that both isoforms contain a PTC before the last intron, a prominent feature of an NMD target that allows for the recruitment of SMG family proteins and subsequent mRNA degradation^20^. It is also worth noting that the presence of a PTC in an NMD target typically occurs at least 55 nucleotides proximal to the downstream exon-exon junction^20,30,31^. DNA sequence analysis of the exons derived from differential splicing of the intron 9 shows that p53β and p53γ have 100 and 12 nucleotides, respectively, between their PTC and downstream exon-exon junction (Fig. 2a). Based on the canonical “55-bp rule”^30^, p53γ is not likely a target of the NMD pathway; however, exceptions have been noted in the case of TCRβ^37^. To further clarify whether the “55-bp rule” contributes to the differential regulation of p53β and p53γ by NMD, we developed a quantitative NMD reporter assay^35^ for p53β and p53γ by cloning the *TP53* genomic DNA region spanning from the intron-9-derived exon to the last exon 11 in frame with the *Renilla luciferase* gene (Fig. 2a, Supplementary Table 2). In these assays, the β and γ reporters mainly differ in the distance from their PTC to downstream exon-exon junction. To validate this approach, we first examined RNA splicing by RT-PCR using a 5′ primer (P1) in the *Renilla luciferase* gene and a 3′ primer located in the *TP53* exon 11 (P2). As shown in Fig. 2b, a single DNA fragment corresponding to the completely spliced β (~905 bp) or γ (~832 bp) form was amplified only from cells transfected with either p53β or p53γ construct. The precise splicing of intron 9 and 10 in the β or γ reporter was further confirmed by DNA sequencing (Supplementary Fig. S2). To examine the reporter genes, we expressed them in wild-type and *SMG7-*null HCT116 cells and found that loss of *SMG7* resulted in a 3-fold increase in the luciferase activities from the p53β reporter and had a lesser effect on the p53γ reporter (~1.5 fold) (Fig. 2c). These data, together with our endogenous findings, suggest that in accordance with typical NMD target hallmarks, p53β, but not p53γ, is the major target of the SMG7-mediated NMD.

### UPF1 depletion induces p53β but not p53γ

**Figure 3:**
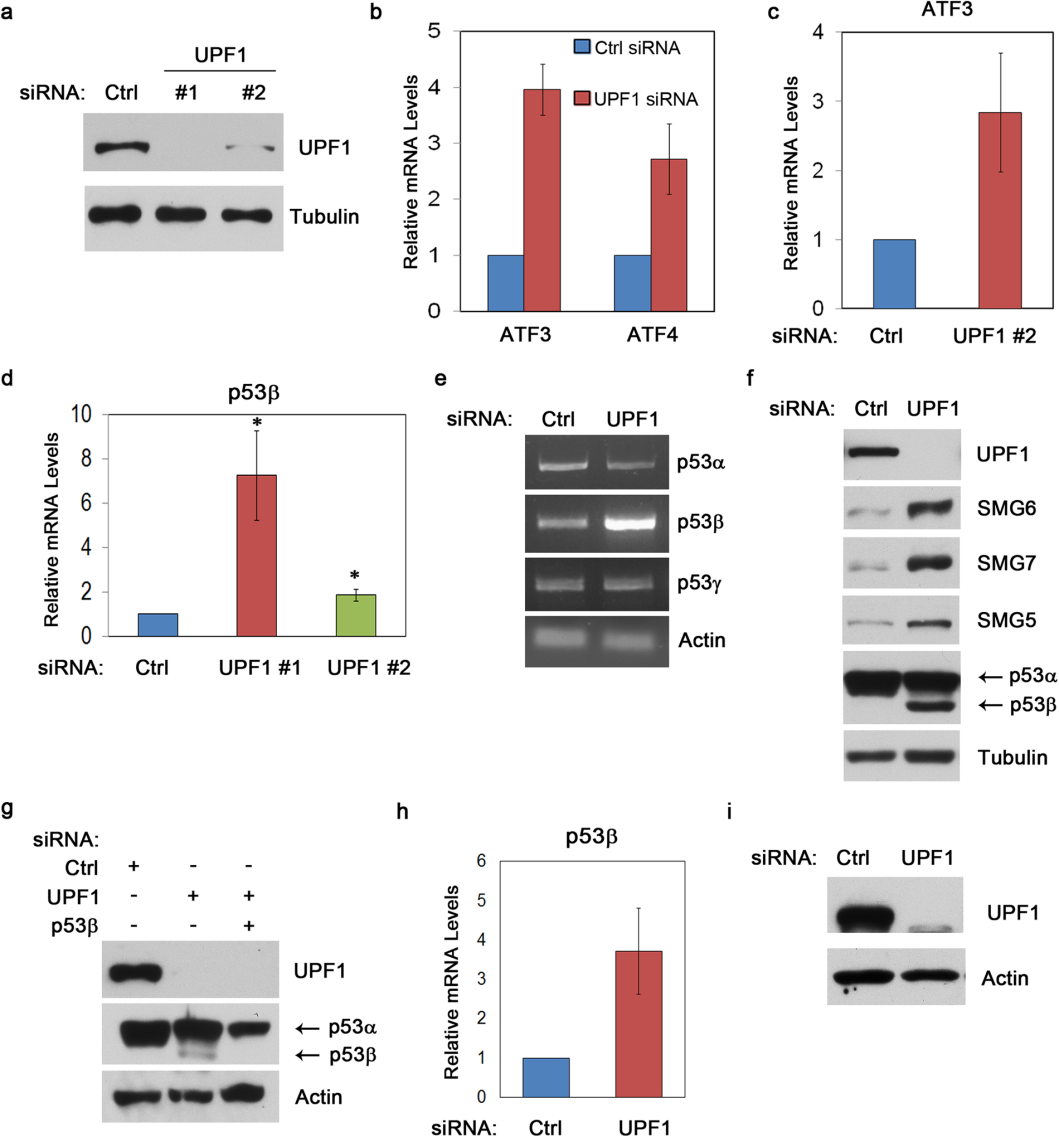
UPF1 depletion induces p53β but not p53γ. **(a)** Western blot analysis of siRNA efficiencies of two independent UPF1 siRNAs (Supplementary Table 1). **(b)** qPCR analysis of known NMD targets (ATF3, and ATF4) in U2OS cells transfected with control (Negative Control #1, Sigma) or UPF1 siRNA #1 and **(c)** UPF1 siRNA #2 (Supplemental Table 1,^22^) for confirmation of NMD deficiency, (n = 3). **(d)** qPCR analysis of p53β isoform mRNA in U2OS cells transfected with control or UPF1 siRNA #1 and #2, p = 0.0094, p = 0.0097, (n = 7). **(e)** Nested semi-quantitative PCR of p53 isoforms^36^ in U2OS cells with control or UPF1 siRNA. PCR cycle numbers were determined by linear amplification. **(f)** Characterization of U2OS cells transfected with UPF1 siRNA was carried out via western blot analysis. NMD core factor expression was analyzed using antibodies as indicated (SMG7, SMG6, SMG5, and UPF1). p53 proteins were detected using the DO1 antibody. **(g)** Further confirmation of p53β (labeled with arrow) band via p53β specific siRNA in U2OS cells. **(h)** qPCR analysis of p53β isoform mRNA in WI38 cells transfected with control or UPF1 siRNA #1, (n = 3). **(i)** Western blot analysis of siRNA efficiency in WI38 cells. Statistical analysis was carried out using students two tailed, unpaired, *t*-test; p < 0.05, SEM, (n = 3). Error bars are utilized to represent SEM.

While the NMD pathway utilizes many different factors, including the SMG family members, and mechanisms in target recognition and degradation, UPF1 appears to be the key and central regulator^28^. To gain more insight into the regulation of p53β by NMD, we examined the p53 isoforms in the UPF1-deficient cells. To this end, we transfected HCT116 cells with UPF1-specific siRNA but failed to obtain efficient knockdown of UPF1 as a result of low transfection efficiency. Thus, we utilized the human osteosarcoma U2OS cells as they provided the best knockdown efficiency, with two independent UPF1-specific siRNAs (Supplementary Table 1,^22,38^) and showed that UPF1 was markedly reduced by both siRNAs (Fig. 3a). As expected, depletion of UPF1 strongly inhibited cellular NMD activities, which was indicated by the induction of two NMD targets ATF3 and ATF4 (Fig. 3b,c). Interestingly, p53β was significantly induced in the UPF1-deficient cells and we noted a robust induction (~7 fold) of p53β when UPF1 was reduced to an undetectable level (Fig. 3d). Using semi-quantitative PCR, we further showed that depletion of UPF1 dramatically increased levels of the p53β full-length transcripts but had minimal effect on p53γ (Fig. 3e), indicating that p53β, but not p53γ, is a major target of the UPF1-mediated NMD. In addition, by western blot analysis we found that the p53β protein, although not detectable in the wild-type cells, was strongly induced in the UPF1-depleted cells (Fig. 3f,g). Notably, several NMD factors were also markedly induced by UPF1 knockdown, which is consistent with previous studies^39^. Furthermore, we made similar observations in the WI38 cells transfected with UPF1-specific siRNA (Fig. 3h,i). Thus, these data demonstrate that the NMD pathway is a critical regulator for p53β.

### NMD determines the fate of p53β following its splicing

**Figure 4:**
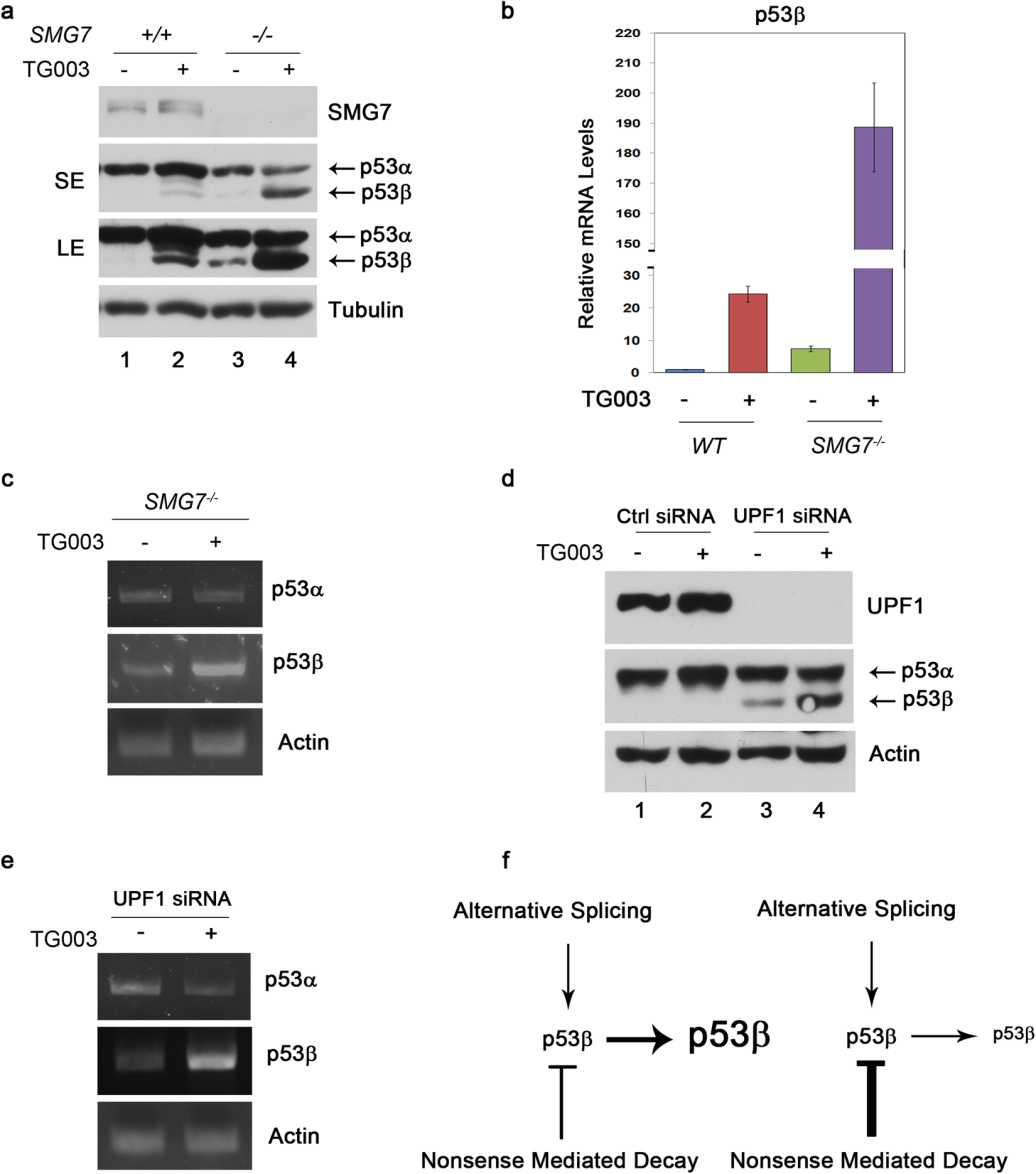
NMD determines the fate of p53β following its splicing. **(a)** Western blot analysis of p53 proteins in HCT116 WT and *SMG7* ^−/−^ cells treated with TG003. **(b)** qPCR analysis of p53β mRNA levels in HCT116 WT and *SMG7* ^−/−^ cells following treatment with TG003 (n = 10). **(c)** Nested semi-quantitative PCR of p53 isoforms^36^ in HCT116 *SMG7* ^−/−^ cells treated with or without TG003. **(d)** Western blot analysis of p53 proteins in U2OS cells transfected with Ctrl or UPF1 #1 siRNA and treated with or without TG003. **(e)** Nested semi-quantitative PCR of p53 isoforms^36^ in U2OS cells transfected with UPF1 #1 siRNA before or after treatment with TG003. TG003 treatment conditions for panels **(a**–**e)**: 50 µM TG003 for 24 hours; Control cells were treated with equivalent volumes of solvent (DMSO). **(f)** A working model depicting the synergistic regulation of p53β by alternative splicing and NMD: the relative activities of NMD and alternative splicing in cells may determine the levels of p53β.

p53β is normally expressed at very low levels, but it can be upregulated under conditions that favor its splicing. Indeed, several splicing factors including SRSF1^18^, SRSF3^17^ and SRSF7^19^ have been identified to modulate p53β expression. As our data show that p53β is tightly controlled by NMD, we investigated how splicing and NMD may regulate p53β in a collaborative manner. To manipulate p53β splicing, we treated cells with TG003, a Cdc2-like kinase (clk) inhibitor that can upregulate p53β by modulating SRSF1-mediated splicing^18^. As expected, treatment with TG003 induced p53β in HCT116 cells at both mRNA and protein levels (Fig. 4a, lane 1 vs 2 and Fig. 4b). Interestingly, the effect of TG003 on p53β was dramatically enhanced by loss of SMG7 (Fig. 4a, lane 2 vs 4 and Fig. 4b) and similar observations were also made when we compared p53β levels in the *SMG7*-null cells before and after TG003 treatment (Fig. 4a, lane 3 vs 4 and Fig. 4c). To examine whether the synergistic regulation of p53β by alternative splicing and NMD observed in the *SMG7*-null cells is a general phenomenon, we assessed p53β expression in U2OS cells following treatment with the UPF1-specific siRNA and TG003. As shown in Fig. 4d, depletion of UPF1 strongly induced p53β (lane 1 vs 3) and, importantly, TG003 treatment further enhanced p53β expression in the UPF1-deficient cells at both protein levels (lane 3 vs 4) and mRNA levels (Fig. 4E). Thus, these data indicate that alternative splicing and NMD play a critical role in their collaborative regulation of p53β expression.

## Discussion

In summary, here we show that the nonsense-mediated mRNA decay pathway plays a crucial role in the regulation of p53β, as demonstrated by disruption of two core NMD factors SMG7 and UPF1. One major function for NMD is to serve as an important quality control mechanism for elimination of aberrant mRNAs containing PTCs, including those derived from alternative splicing^20^. Intriguingly, inhibition of NMD by somatic knockout of *SMG7* or siRNA-mediated knockdown of UPF1 has a major effect on p53β but not p53γ, suggesting that NMD can selectively target PTC-containing alternative transcripts derived from the same gene depending on other factors such as illustrated by the “55 bp rule”^20,30,31^.

As p53β forms as a result of alternative splicing, upregulation of p53β has been observed under conditions that promotes its splicing^15,17–19^. Importantly, our data show that inhibition of NMD further induces p53β following its enhanced splicing, thus illustrating an important interplay between alternative splicing and NMD in the control of p53β expression (Fig. 4f). This working model may suggest that the level of p53β is likely determined by the relative activities of alternative splicing that promotes p53β expression and NMD that degrades p53β. In addition to p53β, human *TP53* gene also encodes three other β isoforms Δ40p53β, Δ133p53β and Δ160p53β, which only differ in their N-terminal region^3^. It is worth noting that Δ133p53β is also induced in *SMG7*-null cells, suggesting that other N-terminal truncated p53 β isoforms can be potentially targeted by NMD, because, like p53β, they all contain the same regulatory elements that are recognized by the NMD machinery. Thus, our findings implicate the necessity to consider the NMD pathway when examining the regulation of p53 β isoforms, for example, in tumors, given that aberrant p53β expression^8–14,40,41^ and dysfunctional NMD^12,22–24^ are both observed in human cancers.

## Methods

### Cell culture

HCT116 cells (wild type; gifted by Dr. Bert Vogelstein) and HCT116 *SMG7* ^−/−^ cell lines were generated in our previous study^34^. HCT116 WT and derivative cell lines were all maintained in McCoy’s 5 A Medium (Cellgro) supplemented with 10% fetal bovine serum (Sigma). U2OS and WI38 cells (ATCC) were maintained in DMEM (Cellgro) supplemented with 10% fetal bovine serum (Sigma).

### Western Blot

Protein was isolated from the cells using Flag Lysis Buffer (50 mM Tris-HCl [pH 7.9], 137 mM NaCl, 10 mM NaF, 1 mM EDTA, 1% Triton X-100, 0.2% sarkosyl, 10% glycerol, and fresh proteinase inhibitor cocktail [Sigma]). Samples (20 µg–40 µg protein) were resolved using SDS-PAGE gel using indicated antibodies. The antibodies used are as follows: UPF1 (Rabbit, Bethyl #A300-036A); SMG5 (Rabbit, Proteintech #12694-1-P); SMG6 (Rabbit, Abcam #AB87539); SMG7 (Rabbit, LS Bio #CPR2435); p53 (Mouse, Santa Cruz DO1 #SC-126); β-actin (mouse, Santa Cruz #SC-47778); Tubulin (Rat, Santa Cruz #sc-53029). All primary antibodies were used in a 1:1,000 dilution. Secondary antibodies: Anti-Rabbit IgG (goat, Cell Signaling #7074); Anti-Rat IgG (goat, Southern Biotech #3050-05); Anti-mouse IgG (Sheep, GE Healthcare #NA931). Secondary dilution ranged from a 1:5,000 to 1:10,000 dilution.

### qRT PCR

Total RNA was extracted from cells using the TRIzoI reagent (Ambion) according to the manufacturer’s protocol. After reverse transcription (MuLV, NEB), the quantitative PCR was performed in triplicate with SYBR Green Master Mix (Applied Biosystems) and the StepOnePlus Real-Time PCR System (Applied Biosystems) with the following PCR conditions: 10 min. at 95 °C followed by 40 cycles of 95 °C for 15 sec. and 60 °C for 1 min. Primers used for PCR: β-actin (5′-CCAACCGCGAGAAGATGACC-3′ and 5′-CGTTGGCACAGCCTGGATAGCAACG-3′^34^); p53β (5′-GAGCACTAAGCGAGCACTGCC-3′ and 5′-TTGAAAGCTGGTCTGGTCCTGA-3′^6^), ATF3 (5′-GCCATTGGAGAGCTGTCTTC-3′ and 5′-GGGCCATCTGGAACATAAGA-3′), ATF4 (5′-GACGGAGCGCTTTCCTCTT-3′ and 5′-TCCACAAAATGGACGCTCAC-3′), ARC (5′-AGCGGGACCTGTACCAGAC-3′ and 5′-GCAGGAAACGCTTGAGCTTG-3′) GAPDH (5′-ACAGTTGCCATGTAGACC-3′ and 5′- TTTTTGGTTGAGCACAGG-3′). qPCR analysis of Δ133p53β was carried out using the PCR product from the first stage of nested PCR for specific amplification of Δ133p53 isoforms^36^.

### p53 Isoform PCR

Nested PCR was carried out for the detection of p53 isoforms mRNA following Khoury *et al*.^36^. PCR was carried out using AD2 polymerase (Advantage 2 Polymerase, Clonetec Takara) for amplification of p53 isoforms and β-actin/GAPDH as a control. From this p53 PCR product semi-quantitative PCR reactions were carried out for linear amplification of α, β and γ p53 isoforms. The PCR product was then run on a 1% Agarose gel for semi-quantitative analysis. Standard PCR for detection of abundant p53 isoform mRNA was carried out with AD2 polymerase (Advantage 2 Polymerase, Clonetec Takara) with primers flanking intron 9 region; Forward (~Exon 9) 5′-GAGCACTAAGCGAGCACTGCC-3′, Reverse (Exon 10) 5′-CATCTCGAAGCGCTCACGC-3′.

### Luciferase Reporters

#### NMD Assay

Constructs were obtained from Andreas Kulozik as a generous gift^35^; pCL-Neo β-globin WT Renilla, pCL- Neo β-globin NT39 Mutant Renilla, and pCL- Neo Firefly,^35^. Constructs were all transfected (Lipofectamine 2000, Invitrogen) with the Firefly control construct (and GFP to monitor transfection efficiency), half were also transfected with Renilla β-globin WT and half were also transfected with Renilla β-globin NT39 Mutant. The cells were harvested after 1.5 days for luciferase detection. Luciferase was detected using a (20/20^n^ Luminomenter, Turner Biosystems) and (Dual Luciferase Reporter Assay System, Promega). *Renilla* signals were normalized to firefly controls. Experiments were carried out in triplicate and also performed two biological replicates.

#### p53 Reporter Assay

Constructs were generated using the NMD assay constructs as a backbone. Using the pCL-Neo β-globin WT Renilla construct we first digested with Xho1 restriction enzyme (NEB) and treated with T4 DNA polymerase (NEB) purified via PCR column and digested with Not1 restriction enzyme (NEB) for removal of the β-globin portion of the construct. cDNA from wild type HCT116 cells was used to PCR amplify our p53 isoform fragments using the Phusion Polymerase (Thermo Scientific). P53β forward primer: 5′- GACCAGACCAGCTTTCAAAAAGA-3′, p53γ forward primer: 5′-ATGCTACTTGACTTACGATGGTGTTACT-3′, p53 isoform reverse primer (Not1 digestion site): 5′aaagcggccgcTCAGTCTGAGTCAGGCCCTTCTG-3′. The fragments were ligated using T4 DNA ligase (NEB); in-frame insertion was confirmed via diagnostic restriction enzyme digestion and sequencing. The cells were transfected using Lipofectamine 2000 (Invitrogen) and harvested after 1.5 days for luciferase detection. Luciferase was detected using a (20/20^n^ Luminomenter, Turner Biosystems) and (Dual Luciferase Reporter Assay System, Promega). *Renilla* signals were normalized to firefly controls. Lysate was also collected for generation of cDNA in HCT116 cells for confirmation of appropriate p53 splicing: forward *Renilla* luciferase primer: 5′-GATTGGGGTGCTTGTTTGGC-3′, reverse p53 isoform primer (Not1 digestion site): 5′aaagcggccgcTCAGTCTGAGTCAGGCCCTTCTG-3′; using Advantage 2 Polymerase (Clontec). Experiments were carried out in triplicate and also performed two biological replicates.

### siRNA

Knock down experiments were carried out with siRNA for UFP1, p53β, and control. The siRNAs were transfected into the cells with Lipofecatmine RNAiMax (Invitrogen) according to the manufacturer’s protocol (cell type specifications). The cells were allowed to recover for 2–3 days and a second reverse transfection was carried out using the same conditions. After 3-4 days the cells were isolated for RNA and protein. siRNA duplex sequences: control (siRNA Universal Negative Control #1, Sigma), UPF1A (GAUGCAGUUCCGCUCCACC [dt][dt], Sigma Custom Oligo^22^), UPF1B (AAGAUGCAGUUCCGCUCC [dt][dt], Sigma Custom Oligo^38^), p53β (GGACCAGACCAGCUUU [dt][dt], Sigma Custom Oligo^18^).

### TG003 Treatment

Cells were treated with 50 µM TG003 (Selleckchem, S7320) prepared in DMSO, control samples were treated with equal volumes of DMSO. Cells were treated for 24 hours prior to harvest for western blot or RNA extraction.

### Statistical Analysis

Microsoft Excel software was used for statistical analysis. Student’s *t*-test was used for comparing two samples (p < 0.05, using a two tailed test). Error bars are utilized to represent SEM.

## Electronic supplementary material

Supplementary Information
