# Supplementary material for: Identification of nonsense-mediated mRNA decay pathway as a critical regulator of p53 isoform β
Source: Sci Rep. 2017 Dec 13;7:17535. doi: 10.1038/s41598-017-17283-4 (PMC5727530; doi:10.1038/s41598-017-17283-4)

Identification of nonsense-mediated mRNA decay pathway as a critical regulator of p53 isoform β

Lauren E. Cowen and Yi Tang*

Department of Regenerative and Cancer Cell Biology,

Albany Medical College

47 New Scotland Ave. Albany, NY 12208

***Corresponding Author:**

Yi Tang

Department of Regenerative and Cancer Cell Biology,

Albany Medical College,

47 New Scotland Ave.

Albany, NY 12208,

tangy@amc.edu

Supplementary Figure Legends:

Supplementary Figure 1:

(a) Assessment of the NMD activities in an independent HCT116 *SMG7*-null clone #2 via the NMD luciferase reporter assay[35], (n=6). **(b)** qPCR analysis of p53β mRNA expression in the HCT116 *SMG7*-null clone #2, (n=4). **(c)** Nested semi-quantitative PCR[36] for full length Δ133p53 isoforms in HCT116 wild type and *SMG7*-null clone #1. **(d)** qPCR analysis of Δ133 p53β expression using nested PCR product specific to Δ133 p53 isoforms[36] **(c)** as a template, (n=2).

Supplementary Figure 2:

Following transfection with the p53β and p53γ Renilla constructs, DNA fragments amplified by RT-PCR using primers P1 and P2 (shown in Figure 2a) were DNA sequenced (GeneWiz, Supplementary Table 1) for confirmation of appropriate splicing of p53. Regions surrounding canonical splice sites (Junctions between intron i9/exon 10 and exon 10/exon 11) are outlined.

Supplementary Table 1:

Summary of siRNA sequences and source for all siRNAs used in the study. All siRNAs purchased through Sigma.

Supplementary Table 2:

Summary of primer sequences, and source for all PCR performed in this study. All primers purchased through Invitrogen.

Supplementary Table 1:

siRNA	Sequence	Reference	Company
UPF1 siRNA #1	GAUGCAGUUCCGCUCCACC [dt][dt]	22	Sigma
UPF1 siRNA #2	AAGAUGCAGUUCCGCUCC [dt][dt]	38	Sigma
Control siRNA	Universal Mission 1		Sigma
p53β siRNA	GGACCAGACCAGCUUU [dt][dt]	18	Sigma

Supplementary Table 2:

Primer Name	PCR	Sequence	Purpose	Ref.
β-actin	qRT PCR	5′-CCAACCGCGAGAAGATGACC-3′ and 5′-CGTTGGCACAGCCTGGATAGCAACG-3′	Housekeeping gene for qRT PCR	34
p53β	qRT PCR	5'-GAGCACTAAGCGAGCACTGCC-3' and 5'-TTGAAAGCTGGTCTGGTCCTGA-3'	mRNA Analysis of p53β	6
ATF3	qRT PCR	5'-GCCATTGGAGAGCTGTCTTC-3' and 5'-GGGCCATCTGGAACATAAGA-3'	mRNA analysis of ATF3	
ATF4	qRT PCR	5'-GACGGAGCGCTTTCCTCTT-3' and 5'-TCCACAAAATGGACGCTCAC-3'	mRNA analysis of ATF4	
ARC	qRT PCR	5'-AGCGGGACCTGTACCAGAC-3' and 5'-GCAGGAAACGCTTGAGCTTG-3'	mRNA analysis of ARC	
GAPDH	qRT PCR	5'-ACAGTTGCCATGTAGACC-3' and 5'-TTTTTGGTTGAGCACAGG-3'	Housekeeping gene for PCR	
P53 isoforms	Nested PCR	See Publication	Semi-quantitative PCR	36
P53 isoforms	Stand. PCR	F:(Ex. 9) 5'-GAGCACTAAGCGAGCACTGCC-3', R:(Ex. 10) 5'-CATCTCGAAGCGCTCACGC-3'	Stand. PCR for detection of p53 isoforms	
P53β primer	Cloning	F: 5'- GACCAGACCAGCTTTCAAAAAGA-3'	For generation of p53 reporter assay: p53β	
p53γ primer	Cloning	F: 5'-ATGCTACTTGACTTACGATGGTGTTACT-3'	Cloning p53 reporter construct: p53γ	
p53 isoform (P2 in Figure 2A)	Cloning	R: 5' TCAGTCTGAGTCAGGCCCTTCTG -3'	Cloning p53 reporter construct: p53β/p53γ	
Renilla luciferase (P1 in Figure 2A)	Splicing/ Sequencing	F: 5'-GATTGGGGTGCTTGTTTGGC-3'	Confirmation of splicing with p53 iso. R cloning primer; Sequencing of constructs	

Supplementary Figure 1

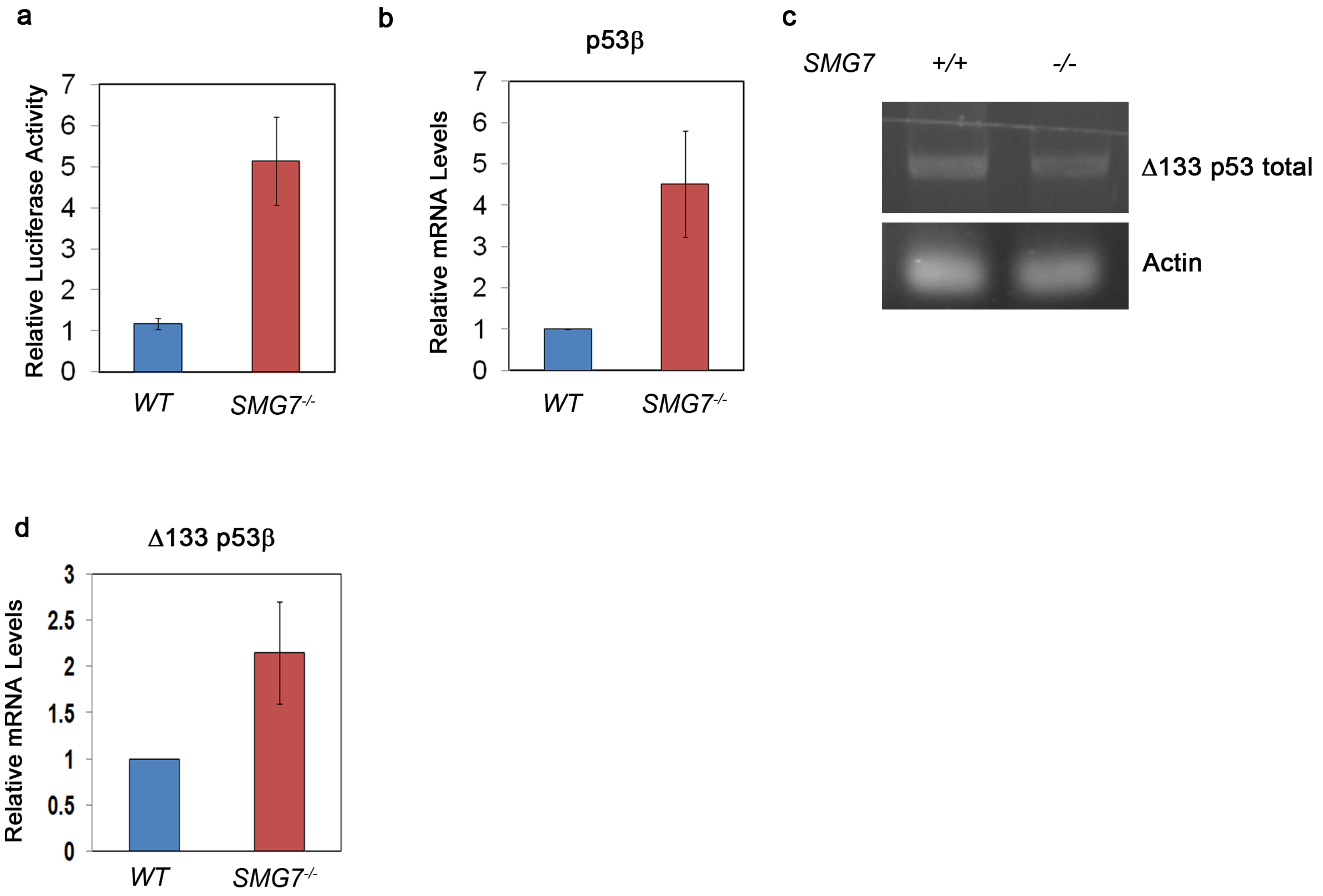

Supplementary Figure 2

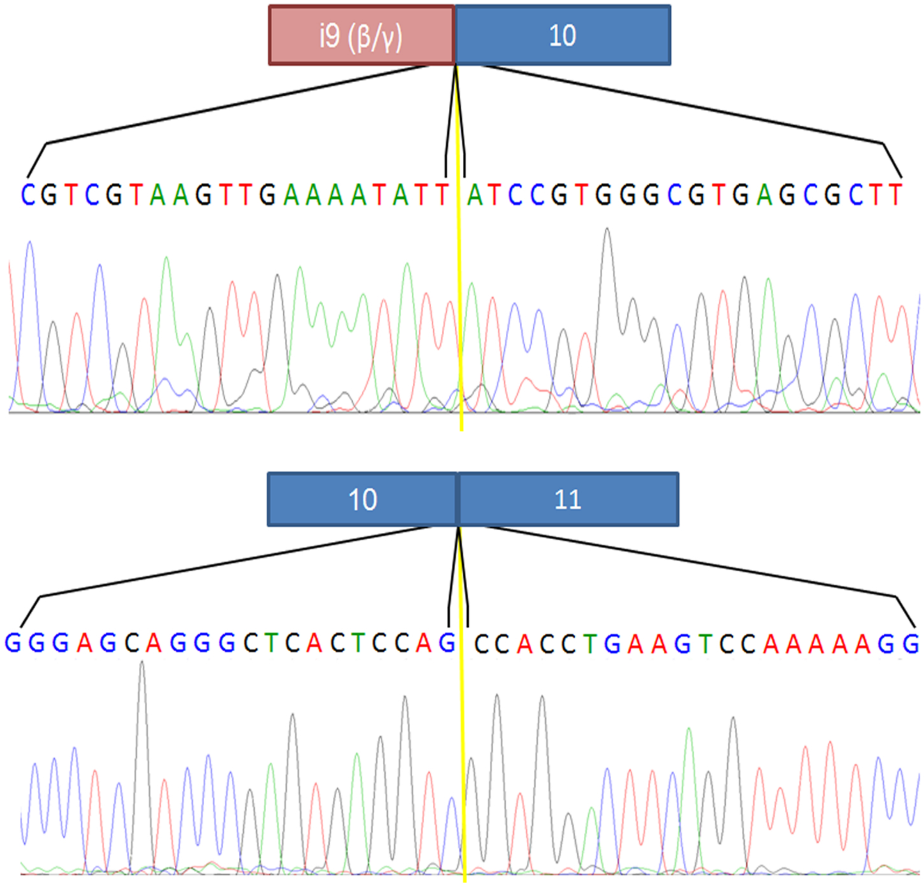

Supplement: Supplementary file 1 — Supplementary Information [file 41598_2017_17283_MOESM1_ESM.pdf]
